# College of Physicians and Surgeons of Philadelphia

**Published:** 1886-01

**Authors:** 


COLLEGE OF PHYSICIANS, OF PHILADELPHIA.

*Stated Meeting held November 4th, 1885.*—The President, Dr. J. M. Da Costa, in the chair.

*Three Cases of Removal of the Ovaries and Fallopian Tubes (Tait's Operation.) By* W. W. Keen, M. D., *Professor of Snrgery, Woman's Medical College of Pennsylvania.*

The following cases are put upon record as a contribution to an important operation, the usefulness of which is assured in certain cases, but the limitations of which have not yet been well defined:

CASE I. *Uterine Myoma; Excessive Hæmorrhage and Anæmia; Tait's Operation; Recovery and Cure.*—Mrs. L., of New Jersey, *æt.* 42; married at twenty-eight; two children—the last born ten years ago; each eleven pounds; normal labors; no miscarriages; absolutely well and strong till three years ago, when her periods became gradually more prolonged and profuse. Now she is unwell three weeks out of every four, and the flow is often so severe as to saturate a napkin every fifteen minutes, besides large clots of blood. She is thoroughly blanched, weak and anæmic.

November 25, 1881. Sent to me for consultation by Dr. Hollingshead. A tumor is visible in the hypogastrium the moment she lies down, and the abdomen is exposed; sound enters 6¼ inches. The tumor is an interstitial myoma in the posterior wall, as large as a large fist, moderately tender and painful; no vegetations on the endometrium; no erosion of os; cervix not involved; uterus moveable. Advised Squibb's extract of ergot (*m* xxx-xl), hypodermically, daily in the abdominal wall for a month; if not then better, advised Tait's operation, as all other means had been previously tried by her attending physician. She was unwell at the end of November, when the ergot was first used. It gave rise to great pain and considerable local inflammation, with nausea and vomiting, and had to be discontinued.

December. Again unwell; the intermenstrual period was freer from pain ; but she was weaker and more blanched, and not able to come to the city; lost a large quantity of blood.

January 26, 1882. Came to the city; was so blanched that had she closed her eyes and folded her waxy hands she could have easily been mistaken for a corpse; weight one hundred pounds. Treatment : iron, quinine, milk-punch; food every two hours.

28th. Taken unwell; period lasted till February 2; used twenty-eight napkins, besides passing a number of large clots. Bad neuralgia of face; morphia (⅛ grain), hypodermically, failed to relieve, but water similarly given lessened it. Eats but little on account of pain.

February 3. Dr. R. P. Harris saw her with me, and concurred in advising the operation. Temp. 98.5°; pulse 80, feeble; heart normal, but weak; no change in uterus.

9th. Operation 12 M. ; antiseptic method with carbolic acid, including the spray; bladder emptied. Duration of operation forty-five minutes. Ether (f℥vijss) used; incision four inches long in median line from pubes half way to umbilicus; no vessels tied.

On opening the abdomen a small amount of serum escaped. On account of the high position of the uterus the ovaries were readily found. Each pedicle was transfixed with a double carbolized silk ligature close to the uterus, the upper including the Fallopian tube, and after ligature the tubes and ovaries were removed. The left ovary showed a recent corpus luteum; it had a few small cysts, and was cirrhosed in part. The right had one cyst two inches in diameter, and several smaller ones. The right tube was cystic just at the cervix-uteri; it contained a serous fluid. The veins were very large; no bleeding requiring a ligature occurred. The ligatures were all cut off short; four deep and two superficial sutures, the former, including the peritoneum, closed the wound. Dressed with carbolized gauze.

Immediately after the operation her pulse was 120, and feeble. Hot-water bottles were applied, and brandy was used, hypodermically, several times with good effect. She vomited only once up to 3.30 P. M., when her pulse was 93; temp. 97.2°; very small quantity of food and stimulant every twenty minutes. 7 P. M., pulse, 100; temp. 99.6°; has had some pain; feels stronger. 11 P. M., temp. 100.8°.

10th. Slept but little, but is comforable; temp. 100°.

11th. Temp. 98.4°; considerable pain in the back at 9 P. M., last night, followed by a bloody vaginal discharge. In twenty-four hours has used sixteen napkins, moderately saturated. Water at 105°–110° ordered, which gave great relief.

12th. Has used twelve napkins; temp. 98.8°.

13th. Slept excellently; discharge has ceased; dressing changed (fourth day). It was barely soiled, with very slight oozing from the operation; no pus; wound free from blush; union by first intention throughout; meat allowed.

15th. Several enemata having had no effect, as she felt uncomfortable, the rectum was emptied, mechanically, of a large amount of impacted scybala.

19th. Redressed; wound healed; sutures removed.

22nd. Sat up.

25th. The menstrual period was due on the 24th. Has used two napkins to date; less than f℥ss blood on each.

March 17. Went home; weight 110 pounds.

24th. Menstruation due; had some backache; no blood; stayed abed three days.

June 6. Came to see me; brown as a berry; weight 130 pounds; appetite good; strength nearly regained; each month had had slight malaise; no bleeding; uterine cavity three and three-quarter inches; myoma not perceptible, except by bimanual examination.

March, 1884. Rapidly regained full strength; weight has continued at 140 pounds; no bleeding; sexual appetite unimpaired; tumor entirely gone; uterus three inches.

CASE II. *Severe Nymphomania, leading to Incipient Insanity; Menorrhagia; Tait's Operation; Recovery; Cure.*—Mrs. B., of New Jersey, *æt.* 42, American; eight children—last born eight years ago; operated on by me, successfully, four years ago for lacerated perineum, and later, another operation for severe hemorrhoids. Wife of a poor, ill-paid clergyman, and hence her life was a constant struggle properly to feed and clothe her large family. I have known her from childhood. She was always a most exemplary Christian woman.

Her head began to trouble her not long after the first operation, and she attributed it to the ether, which, however, she bore perfectly well in both operations. She had strange feelings as if unconscious, and in fright or dread of ether, especially at night. Exposed to the sun, in August, 1881, she had an attack of heat-exhaustion, followed by a second attack a week later. After this her menstruation, always previously easy and regular, ceased for three months. During this time she was treated for malaria, and her head became worse, which she attributed to the quinine. She became very nervous and sleepless; lost all self-control; could not bear any noise of the children, the church-bell, or even her own voice. She became unable to do any work, and had extreme depression of spirits; attempts at suicide were repeatedly contemplated, and though almost determined to end her life, she was deterred by her religious fears. These emotions were readily confessed to me and to her husband. In December, 1882, her menstruation became very profuse, and was continuous for three months. Since then it is not continuous, but is still very profuse.

Meantime, in October, 1881, by spells her sexual appetite, till then a matter of little moment, became immoderate. Day and night it was an exquisite physical and mental torment, and even led her to repeated self-abuse when it could not be gratified. This nymphomania and her head symptoms were always worst at her menstrual period. Finally, she went voluntarily to an insane hospital, in March, 1882, being utterly unfitted for her household duties, and in constant dread of suicide; but soon returned home.

*January* 2, 1883. I saw her; head still as described, and she was almost desperate; uterus normal, except some erosion at os, and freely movable; clitoris and other generative organs normal. Her attacks of nymphomania were still frequent and severe, especially during menstruation. She was fast passing toward permanent insanity. She loathed herself for her abnormal sexual appetite; she had struggled against it, as well as against her suicidal intent, till she was ready to hail anything that gave the faintest hope of relief at any risk of life, for which she cared absolutely nothing. She had been under varied and excellent care, and every moral means and all promising drugs had been freely tried. I therefore proposed Tait's operation, to which she and her husband instantly assented.

4th. Operation ; ether ; antiseptic method (carbolic acid), with spray; bladder emptied; incision three inches in median line upwards from pubes ; layer of fat (she was well nourished) one inch thick, belly-wall two inches. Left ovary found without difficulty ; its pedicle pierced by needle with eye in the point carrying a double carbolized silk ligature ; ovary and Fallopian tube tied separately and ligatures cut short. One ovarian vein was varicose and as large as the little finger ; ovary and tube both removed, Two pedunculated growths of the size of peas were attached to the ovary, one directly and another from the middle of a long foot-stalk attached at the two ends to the ovary and to the tissue between the ovary and tube. The right ovary was found with some little difficulty ; as it was pulled out of the wound a small cyst burst. It was treated precisely as the left, and tube and ovaries removed. Both tubes and ovaries were intensely congested (her last menstruation was five days past) ; several small cysts existed in each.

Her recovery was uninterrupted. She had a little bilious vomiting and retention of urine requiring the catheter, but no pain ; and no medicine.

8th. A moderate vaginal hæmorrhage began, which ceased four days later spontaneously.

9th, 11th, and 14th. The stitches were removed. Her highest temp. was 99.4°.

19th. Down stairs.

23d. Went home. Since then I have seen her repeatedly ; the last time in the spring of 1885. Her mental symptoms and head troubles have gradually become better. For the first six months or more she was often despondent, but she gradually recovered her cheerfulness to a large extent, resumed her household occupations, and is perfectly well. The nymphomania ceased from the time of the operation, save two very slight and short attacks. Coitus is rare, but is entirely normal, and is not followed by any tendency to her former deplorable condition.

CASE III. *Uterine Myoma; Severe and Long-continued Hæmorrhage; Operation; Death.*—Miss W., *æt.* 40, first menstruated at fourteen, always profusely. For the last seven to eight years much worse, the flow continuing ten to fourteen days. In May, 1884, she began to suffer from continuous hæmorrhage, which has persisted till the present date, January 2, 1885. Occasional severe hæmorrhages also occurred. She is very pale and anæmic, with waxy lips, and has lost much flesh and strength, especially of late. To-day I examined her under ether: uterus three inches in length, and movable; a myoma as large as the fist was discovered in the anterior wall and fundus. Hypodermatic injections of Squibb's ergot, in f℥j doses, every second day, were used, to which, later, was added f℥j of the fluid extract of ergot daily, with tonics and good diet.

January 29th. Has passed the menstrual period without noticeable hæmorrhage, and to-day, for the first time since last May (excepting two days), has dispensed with a napkin. From this date till April her menstruation ceased. In April and May she had a normal discharge. But in June the hæmorrhage returned, and continued so profusely as to threaten life.

July 4th, 1885. The hæmorrhage having been checked for three days by the above means, I operated. The tumor, which had clearly increased in size, was immediately seen on uncovering the belly. Ether; antiseptic precautions, including the spray (carbolic acid); bladder emptied. The enlarged uterus was so much in the way that the ovaries could not be seized through the small incision first made in the linea alba, the ovaries not having been carried up with it, and it had to be prolonged one inch above the umbilicus. The whole hand had to be introduced, the uterus lifted and pushed forcibly aside, and the ovaries were even then reached with the greatest difficulty, and after several attempts. The ovary and tube on each side were removed, the pedicle being tied with stout carbolized silk, which was cut off short.

The left tube was attached to the ovary at the fimbriated extremity; two cysts, one filled with blood and one with serous fluid, existed in this ovary, the stroma of which was largely cirrhosed. One large (size of English walnut) and one smaller serous cyst were found in the right ovary, and its stroma was atrophied and cirrhosed. All four cysts were ruptured during removal. About eight ounces of serum were found in the peritoneum.

5th. The wound was united with silver wire sutures after careful cleansing of the peritoneal cavity (there was no bleeding), and then dressed with carbolized gauze. Symptoms of peritonitis began to develop, and in spite of all remedies progressed to a fatal issue on July 7. The temperature was 102°-103° till shortly before death, when it rose to 106°.

Autopsy, July 8th. Recent lymph was found over a considerable portion of the belly contents, with an ounce of pus in Douglas's *cul-de-sac*. No hæmorrhage had occurred.

